# Absolute and proportional measures of potential markers of rehearsal, and their implications for accounts of its development

**DOI:** 10.3389/fpsyg.2015.00299

**Published:** 2015-03-23

**Authors:** Christopher Jarrold, Henrik Danielsson, Xiaoli Wang

**Affiliations:** ^1^School of Experimental Psychology, University of Bristol, BristolUK; ^2^Linköping University, LinköpingSweden; ^3^The Swedish Institute for Disability Research, LinköpingSweden; ^4^North West Normal University, LanzhouChina

**Keywords:** rehearsal, proportional scaling, phonological similarity effect, word length effect, development

## Abstract

Previous studies of the development of phonological similarity and word length effects in children have shown that these effects are small or absent in young children, particularly when measured using visual presentation of the memoranda. This has often been taken as support for the view that young children do not rehearse. The current paper builds on recent evidence that instead suggests that absent phonological similarity and word length effects in young children reflects the same proportional cost of these effects in children of all ages. Our aims are to explore the conditions under which this proportional scaling account can reproduce existing developmental data, and in turn suggest ways that future studies might measure and model phonological similarity and word length effects in children. To that end, we first fit a single mathematical function through previously reported data that simultaneously captures absent and negative proportional effects of phonological similarity in young children plus constant proportional similarity effects in older children. This developmental function therefore provides the benchmark that we seek to re-produce in a series of subsequent simulations that test the proportional scaling account. These simulations reproduce the developmental function well, provided that they take into account the influence of floor effects and of measurement error. Our simulations suggest that future empirical studies examining these effects in the context of the development of rehearsal need to take into account proportional scaling. They also provide a demonstration of how proportional costs can be explored, and of the possible developmental functions associated with such an analysis.

## Introduction

Many models of verbal short-term memory (e.g., Baddeley, 1986; Camos et al., 2009), assume that individuals maintain to-be-remembered verbal material via a process of subvocal rehearsal. Indeed, the phonological loop component of Baddeley’s working memory model consists of two sub-components – a phonological store that maintains phonological representations, coupled with a rehearsal process that offsets the degradation of these representations that would otherwise be caused by trace decay. As a consequence, the extent of forgetting from verbal short-term memory is assumed to depend on the efficiency of rehearsal. Specifically, the faster an individual can rehearse an item the less that item suffers from forgetting (Schweickert and Boruff, 1986). This provides a potential explanation for the word length effect – the finding that adults show lower short-term memory spans for words of a long as opposed to a short spoken duration (Baddeley et al., 1975) – and in turn the word length effect has therefore been viewed by many as a marker of rehearsal taking place.

A second potential indicator of rehearsal is the presence of a phonological similarity effect for visually presented material. The phonological similarity effect is the well-established finding of poorer recall from verbal short-term memory of sets of phonologically similar compared to phonologically dissimilar items (e.g., Wicklegren, 1965). This effect is assumed to reflect the confusability of similar items within the phonological store of Baddeley’s model. However, when observed for visually presented material it also shows that the participant has recoded this visual information into a phonological form. Because this process of recoding involves naming the stimuli internally, it has been argued that participants who can recode in this way can also rehearse (e.g., Howard and Franklin, 1990). Indeed, in the neuropsychological literature the absence of a phonological similarity effect for visually presented material is typically interpreted as evidence of a failure to recode and rehearse (see Trojano and Grossi, 1995).

Since the seminal work of Flavell et al. (1966), developmental psychologists have assumed that the process of subvocal rehearsal takes time to come on-line, and is absent in younger children. Evidence from studies of the two potential markers of rehearsal identified above appears to support this view. Children aged younger than 7 years often fail to show significant word length effects. This is shown in their performance on verbal short-term memory tasks in which lists of items with verbal labels of either a short or a long spoken duration are presented for immediate serial recall (Allik and Siegel, 1976; Henry, 1991; Henry et al., 2000; Turner et al., 2000). Children of this age similarly tend not to show reliable phonological similarity effects for visually presented material. In such studies children are typically shown pictures of objects that either do or do not have phonological similar names (e.g., dog, man, tree vs. cat, hat, mat), and older, but not younger children, show poorer recall or lower immediate memory spans for phonologically similar lists (Hitch et al., 1989, 1991; Halliday et al., 1990; Palmer, 2000). Indeed, a recent study by Henry et al. (2012) that simultaneously looked at both word length and phonological similarity effects for visually presented material, either by measuring memory spans or proportion of items recalled from lists of a fixed length, failed to find either effect in children aged below 6. Consequently Henry et al. (2012) argued that rehearsal is absent at these young ages, and others have similarly claimed that children do not engage in rehearsal until around 7 years of age (Baddeley et al., 1998; Gathercole, 1998; Jarrold et al., 2000).

Although widely held, recent evidence has challenged the assumption that rehearsal undergoes a qualitative change around this age (see Jarrold and Hall, 2013). For example, Jarrold and Citroën (2013) replicated previous studies in showing that the absolute size of the phonological similarity effect increased with age between 5 and 9 years, and was absent in the youngest children with visual presentation of material. However, when the size of this effect was coded as a proportion of individuals’ ‘baseline’ performance (i.e., [dissimilar recall – similar recall]/dissimilar recall), the majority of these developmental differences were removed, with participants of all ages showing a comparable proportional cost of similarity (cf. Logie et al., 1996; Beaman et al., 2008). This suggests that apparent developments in the size of this effect with age are in fact a consequence of proportional scaling operating across different baseline levels of performance, rather than necessarily reflecting a qualitative change in use of rehearsal with age.

Having said this in the Jarrold and Citroën (2013) data the observed phonological similarity effect was sometimes even smaller than would be predicted by this proportional scaling account. Specifically, this occurred in just one cell of the design; namely among the youngest children in the condition where material was presented visually and then recalled verbally. In fact this condition produced the lowest baseline (dissimilar) recall among all individuals, raising the possibility that floor effects among the youngest participants reduced the similarity effect more than would be expected by proportional scaling. If there is a lower bound to the possible range of recall of phonologically similar items, then the observed level of recall of such items could conceivably be greater than that predicted by proportional scaling. In addition, noise in the measurement of recall of phonologically similar items could serve to increase the observed value of similar recall above that predicted by proportional scaling. However, if performance is near floor then noise cannot reduce similar recall below this floor level. Subsequent modeling of the data from the visual presentation and verbal recall condition supported these suggestions, leading Jarrold and Citroën (2013) to argue that the phonological similarity effect (and by implication the word length effect) would be expected to scale in proportion to children’s level of performance, except in conditions where recall was close to floor.

The purpose of the current paper is not to provide further evidence for the proportional scaling account of phonological similarity and word length effects, but rather to explore the implications of this view and the boundary conditions under which it can and cannot provide a good account of developmental data. We have two specific aims. The first is to examine what, if any, additional assumptions have to be added to a proportional scaling model to reproduce the developmental data. We do this in a series of mathematical simulations that start with the simple assumption of proportional scaling of these effects, and which then go on to successively add additional assumptions. Our second aim is, as a result, to provide an example of how future studies in this area might go about examining their data and testing these assumptions.

The above review shows that the general pattern of developmental data that needs to be reproduced in our simulations is one of absent manipulation effects in young children and larger effects in older individuals. More specifically, the Jarrold and Citroën (2013) data show that when manipulation effects are coded in proportional terms, the majority of developmental differences disappear. However, at very low levels of recall (i.e., particularly among younger children) manipulation effects can be smaller than predicted by proportional scaling and can even be reversed. To provide a clear benchmark against which to evaluate our simulations, we first fit a mathematical model through the Jarrold and Citroën (2013) data to provide a single developmental function that the simulations should re-produce.

### Fitting a single function to the Jarrold and Citroën (2013) dataset

Jarrold and Citroën (2013) assessed a total of 117 children aged between 5 and 9 years on immediate serial recall tasks that required each individual to remember either phonologically dissimilar or phonologically similar items. Children were allocated to one of four separate testing conditions that were formed by crossing the modality of presentation of the memoranda (auditory vs. visual) with the modality in which recall was required (spoken vs. pointing to a set of response pictures). Recall of phonologically dissimilar and similar items was assessed in separate blocks, and each block involved the presentation of four trials at a series of increasing list lengths. Testing started at list length 2, and proceeded to the next list length if the participant recalled all the items presented on a trial in their correct serial order on at least one occasion. The dependent measure extracted from each block was a partial credit unit scores for performance across all trials in the block, rather than span scores (see Conway et al., 2005). This measure sums the proportion of items on a trial that are correctly recalled across all trials that a child was presented with. In other words, a child who correctly recalled all the items on all trials at list lengths 2 and 3, but only two of the four items on all trials at list length 4, would receive a partial credit unit score of 10 [(4 ^∗^ 1) + (4 ^∗^ 1) + (4 ^∗^ 0.5)].

**FIGURE 1:**
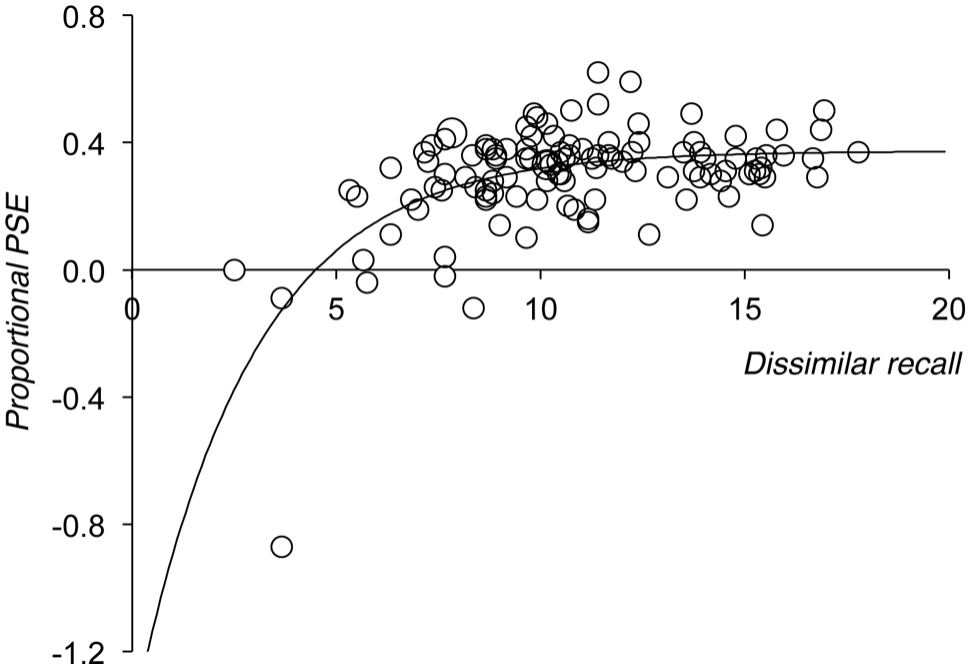
**Proportional size of the phonological similarity effect, plotted against individuals level of dissimilar recall, for all participants in the Jarrold and Citroën (2013) study.** Overlapping data are represented by proportionally larger points. Dissimilar recall is measured in terms of partial credit unit scores (Conway et al., 2005; see Jarrold and Citroën, 2013).

**Figure 1** replots the proportional size of the phonological similarity effect shown by each participant in the Jarrold and Citroën (2013) study against that individual’s ‘baseline’ level of recall. Baseline level of recall was indexed by their partial credit unit score for phonologically dissimilar items. The graph shows that at higher levels of baseline recall proportionalised phonological similarity effects are positive, but that negative proportional phonological similarity effects are seen at lower levels of baseline performance. This developmental pattern was modeled using a negative exponential growth function, see equation 1.

$$\mathrm{Pr}oportional\,\;effect=A+\left(B*\left(1-e^{-c*baseline\,\;\;recall}\right)\right)$$

This kind of function is consistent with the fact that the data show negative proportional scores at low levels of baseline recall (hence one would expect the intercept value, A, to be negative), and scores that tend to aggregate around a constant value when baseline recall is higher (the asymptotic proportional effect score is given by A + B; C represents the rate of growth of the function).

The best fitting negative exponential growth function, shown in **Figure 1**, was fit using the non-linear regression module of SPSS, and accounted for a significant proportion of the variance in the proportionalised effect (*R*^2^ = 0.36). The resultant parameters showed that the function leveled off to an asymptotic value of 0.37. In other words, according to this analysis, a relatively constant 37% cost of phonological similarity was observed in this study once baseline dissimilar recall scores were above a certain level (the function predicts a 35% cost when baseline scores reach a partial credit score of 13, and a 37% cost for partial credit scores of 18 and above).

### Simulations

In all the simulations reported below we look at the effects of a hypothetical manipulation (which could correspond to a manipulation of word length or phonological similarity) in terms of the difference between a baseline condition (which would correspond to a short word or a phonologically dissimilar condition) and a harder manipulation condition (which would correspond to a long word or phonological similar word condition). We plot the size of the manipulation against baseline recall in order to model the effects of development, assuming that age would be associated with increases in baseline levels of recall. In most simulations we plot the size of the manipulation in two ways; the absolute size of the effect (baseline – referent) and the proportionalised effect ([baseline – referent]/baseline). The former allows us to present the data in the manner in which such effects are typically reported in the literature, while the latter allows us to examine whether the simulation produces a function that mirrors seen in **Figure 1**.

#### Simulation 1 – Proportional Scaling without Noise

**FIGURE 2:**
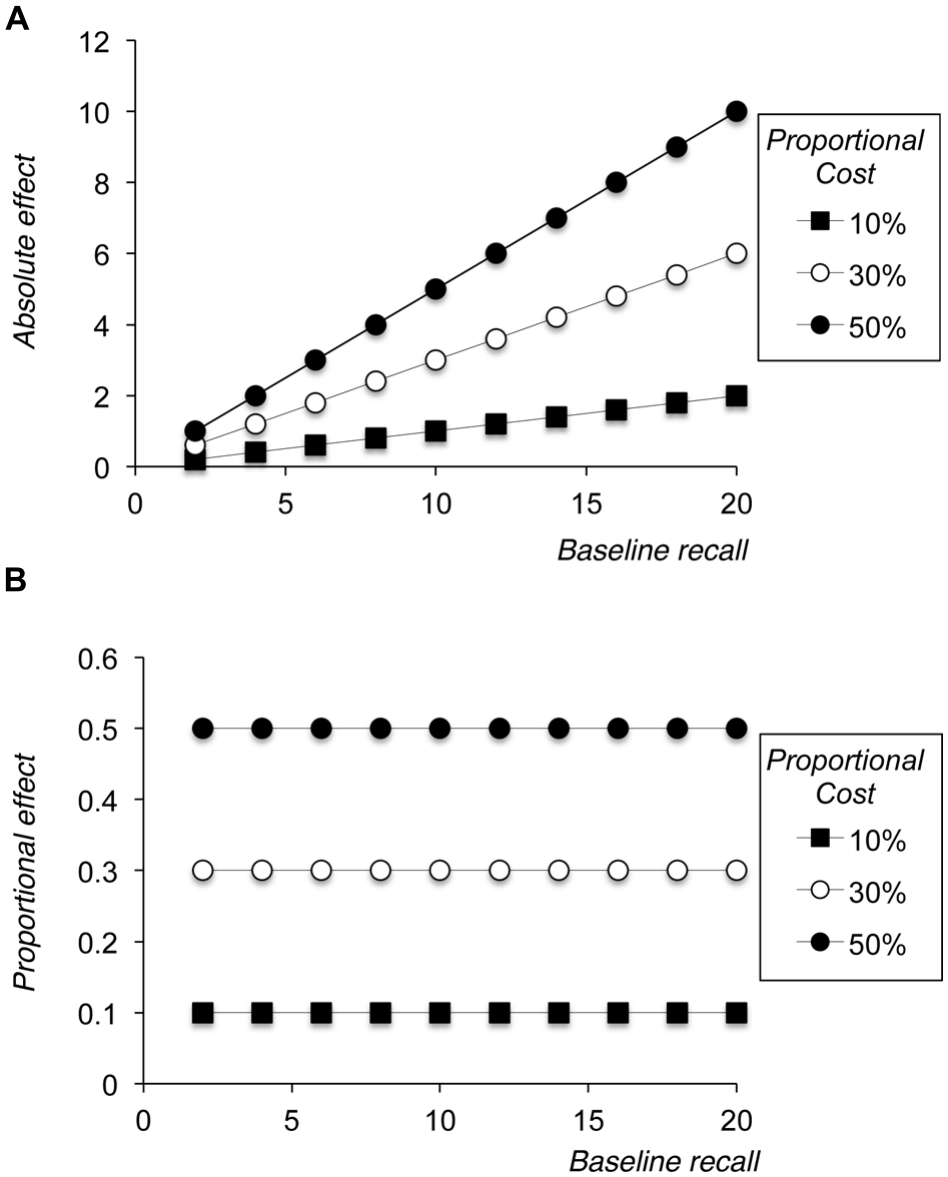
**Results of Simulation 1, showing size of the absolute effect of the manipulation **(A)** or the proportional effect of the manipulation **(B)** plotted against baseline level of performance**.

In Simulation 1 we artificially assumed that there was no noise in the estimate of baseline or harder manipulation condition recall, and employed three different levels of proportional cost of our manipulation – 10, 30, and 50%. **Figure 2** shows the plots that arise from these simulations when the manipulation is measured in absolute terms (panel A) or proportional terms (panel B). Panel A necessarily shows that the absolute size of the effect is linearly related to baseline recall, with a slope that corresponds to the proportional cost; panel B necessarily shows that proportional costs are flat across levels of baseline performance. These results are entirely unsurprising and, indeed, arguably do not need to be demonstrated graphically. However, they serve as a reference point against which one can compare more realistic simulations that include noise in the estimate of the baseline and referent conditions.

#### Simulation 2 – Proportional Scaling with Proportional Noise

In Simulation 2 we assumed that the size of any noise in the estimate of performance in either the baseline or harder manipulation condition was also proportional to baseline level of performance. In other words, we assumed a form of heteroscedasticity where the variance in the underlying population of potential scores for both the baseline and harder manipulation condition at any point in the distribution was proportional to its mean. Specifically, in Simulation 2 we fixed the proportional cost of our manipulation to 30% and simulated 2000 data points (sampling from the distribution of baseline scores between 0 and 20 at 0.01 intervals). For each sampled value of baseline performance, an ‘observed’ baseline score was determined by the addition of noise. This noise was drawn randomly from a Gaussian distribution of potential values that was centered on zero and had a standard deviation that was equivalent to 10% of the sample value of baseline performance for that point. Observed performance in the harder manipulation condition was calculated by first imposing a 30% cost on the sampled (rather than observed) level of baseline performance, and by then adding noise in the same way as for the baseline performance scores. However, the noise for the harder manipulation condition was sampled independently from the sampling of noise for the baseline condition.

**FIGURE 3:**
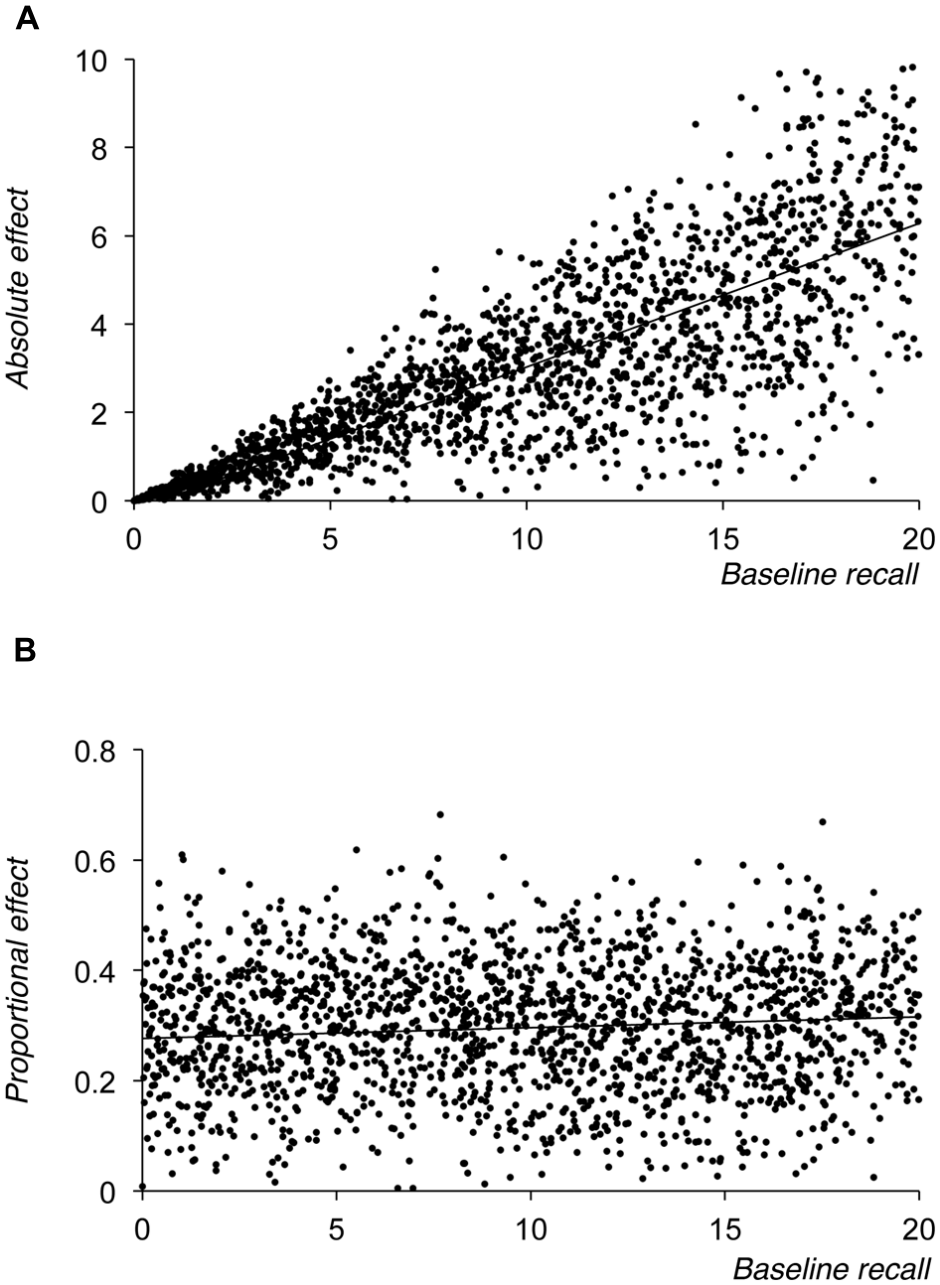
**Results of Simulation 2, showing size of the absolute effect of the manipulation (A) or the proportional effect of the manipulation (B) plotted against baseline level of performance**.

As **Figure 3A** shows, unsurprisingly the plot of absolute manipulation sizes shows a similar pattern to that seen for the 30% cost curve in **Figure 2A**, but with increasing variance in the size of the effect as baseline performance increases. However, when coded proportionally (see **Figure 3B**) the variance in proportionalised manipulation scores does not increase across the range of baseline performance values. This confirms that if one is seeking to examine whether proportional scaling occurs in the development of a manipulation, then plotting proportional scores against baseline performance should produce a relatively flat developmental function even if noise is present, provided that this noise also scales with level of baseline performance.

#### Simulation 3 – Proportional Scaling with Constant Noise

**FIGURE 4:**
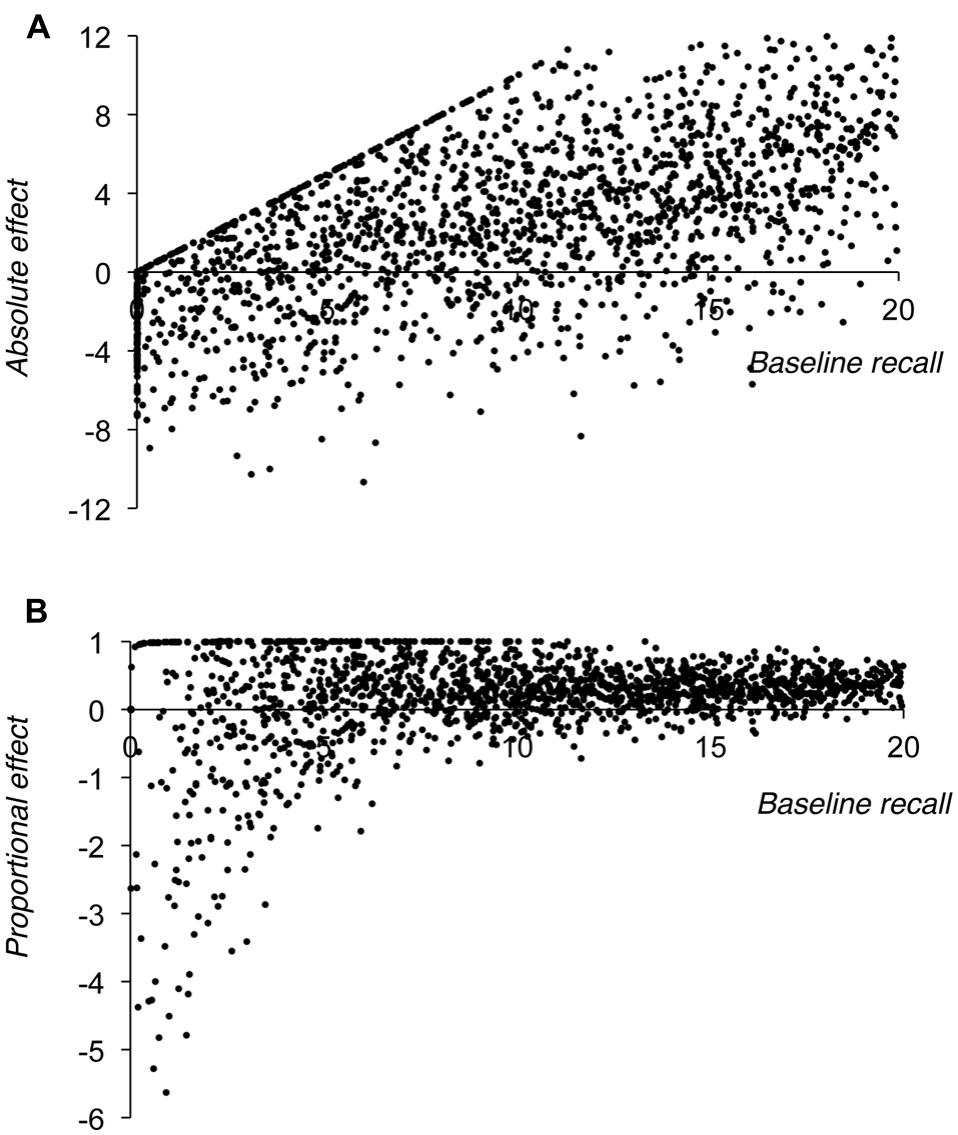
**Results of Simulation 3, showing size of the absolute effect of the manipulation (A) or the proportional effect of the manipulation (B) plotted against baseline level of performance**.

One consequence of assuming in Simulation 2 that noise in the estimates of baseline and harder manipulation condition performance are themselves proportional to level of baseline performance is that this noise tends to zero as baseline performance approaches zero. In Simulation 3 we instead assumed that the spread of the noise distribution in the observed estimates of baseline and harder manipulation condition performance was constant across all levels of sampled baseline performance. On the basis of preliminary simulations we fixed the standard deviation of the noise distribution to three partial credit score items; the noise distribution was again centered on zero. An immediate but unsurprising problem that follows from assuming constant noise is that this produced predicted values for both baseline and harder manipulation condition performance that were, on occasion, below zero, particularly at lower sampled values of baseline performance. Clearly it is not possible for participants to score less than zero and so we elected to raise all such negative observed baseline and harder manipulation condition partial credit scores to zero. In fact, given our interest in proportional effect sizes we raised these scores to 0.01, as a baseline score of zero leads to a non-computable proportional effect. **Figure 4** shows the resultant plots.

**Figure 4A** shows that when the manipulation effects are plotted in absolute terms, the constraint that performance can never drop below zero produces an upper bound to the size of the effect at low levels of baseline recall. This poses problems for any analysis that would seek to fit a linear regression through these data as the resultant slope of this regression would be artificially reduced by these points. Similarly, **Figure 4B** shows two features that follow from the fact that recall can never be negative. First, at lower levels of baseline performance there are a number of points with a proportional score of exactly +1. This follows from the fact that referent condition performance is close to zero for these points (as a result, the proportionalised effect = [baseline score – 0.01]/baseline score, which tends toward 1). This therefore represents a necessary upper limit on the size of the proportionalised manipulation effect. Second, and in contrast, there is necessarily no correspondingly lower limit to these proportional scores. Indeed at low levels of baseline recall there is a preponderance of very large negative proportional scores. These points arise because noise in the estimate of referent condition performance leads to a larger score for this condition than for the baseline condition (i.e., overriding the proportional cost to produce a negative manipulation effect). Large negative proportional scores necessarily arise when negative absolute effects are proportionalised by being divided by small baseline performance values.

#### Simulation 4 – Proportional Scaling with Constant noise – Investigating the Impact of Varying Noise and Floor Effects

Simulation 3 showed that the effect of constant (with level of ability) noise in the estimates of baseline and referent condition performance, coupled with the fact that these scores could never drop below zero, produces a pattern of data that looks broadly similar to that seen in **Figure 1**. In Simulation 4 we therefore further explored the effects of varying the size of constant noise and the level of floor performance, and modeled the resultant data using negative exponential growth functions of the form shown in **Figure 1**. Specifically, SD of noise values of 2, 3, and 4 partial credit score items were examined, again with the noise distribution centered on zero, coupled with floor values of 0 (in fact 0.01), 1, or 2 items. The notion of a functional floor above zero follows from the fact that there are reasons to suspect that recall of zero items is unlikely to be observed in any verbal short-term memory experiment. Indeed, although models of immediate recall sometimes suggest that the core capacity short-term memory is less than the total amount of items recalled on a task (Cowan, 2001; Oberauer, 2002), these models would argue for a focus of attention that, in the limit, would not be expected to ever be less than one item. Furthermore, in any study investigating the phonological similarity effect in children one can only predict a similarity effect on lists that contain at least two items, because by definition similarity depends on the overlap between items in the list. Consequently, individuals might well be expected to recall 1 item even on similar lists.

**FIGURE 5:**
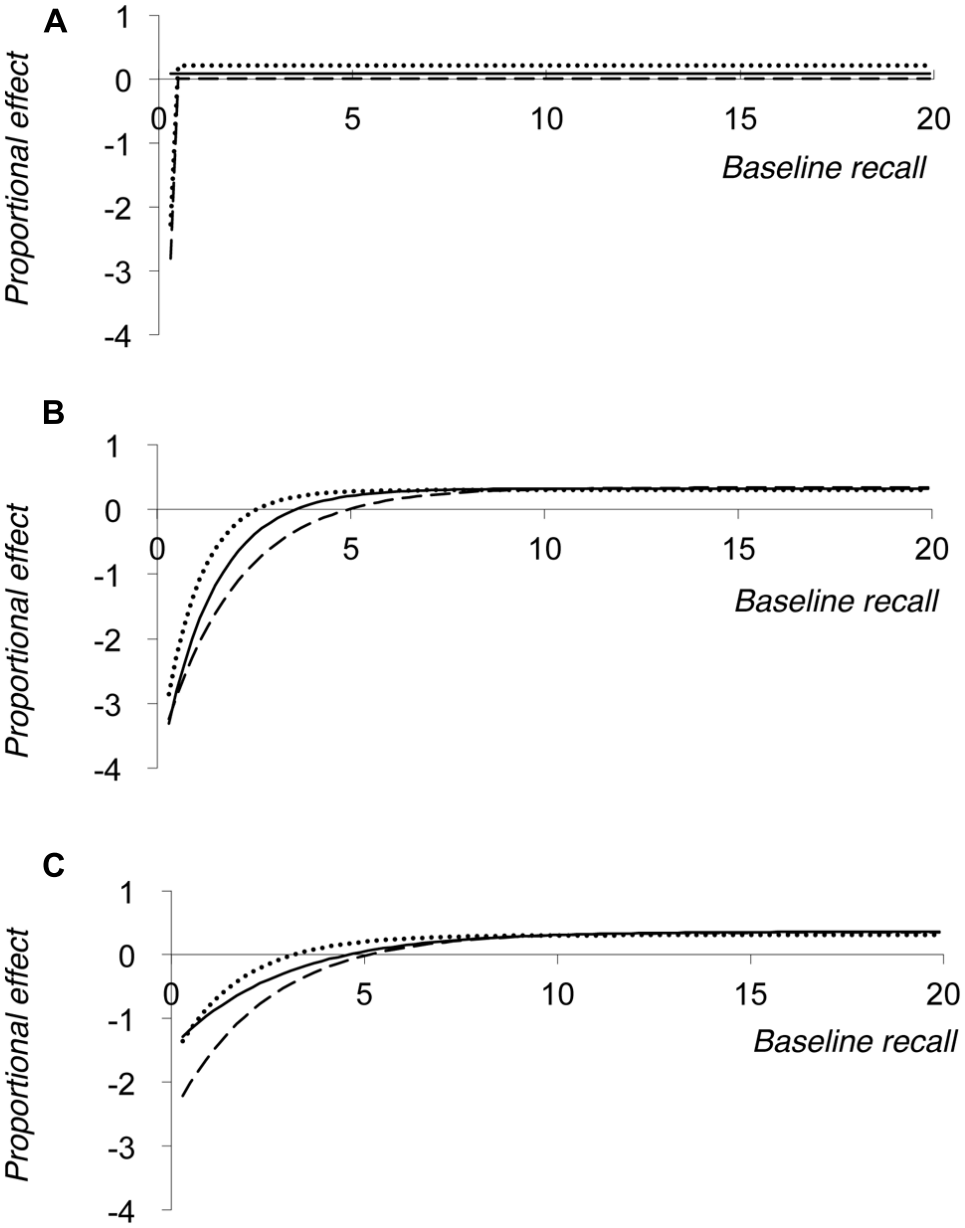
**Results of Simulation 4, showing negative exponential growth functions fit to the simulated data for proportional effect of the manipulation plotted against baseline level of performance.** Functional floor values are set to either 0 **(A)**, 1 **(B)**, or 2 **(C)** partial credit score values, with each panel showing the effect of SDs of noise of 2 (dotted line), 3 (solid line), or 4 (dashed line) partial credit scores.

Simulation 4 therefore produced nine datasets (3 values of noise × 3 values of floor), which are represented in **Figure 5**. Each model contained 2000 data points as in Simulations 2 and 3, although these data points are not shown in the figure (for reasons of clarity). Instead, **Figure 5** plots the results of the negative exponential growth functions that were fitted to the resultant proportionalised effect data sets (note that absolute effects are not plotted in **Figure 5**, in contrast to **Figures 2**–**4**).

The figure shows that when the floor is set at zero (or, more accurately, 0.01 partial credit score points) functions that look broadly similar to that seen in **Figure 1** emerge (see **Figure 5A**), but with a more rapid rise to the asymptotic value and a correspondingly earlier inflection point in the function. In addition, the three different functions plotted in **Figure 5A** produce slightly different asymptotic levels of proportional cost, specifically 0.210, 0.086, and 0.003 for noise of 2, 3, or 4 SDs distribution respectively. This shows that these negative exponential growth functions are not adequately capturing the data, as all three models should converge on an asymptotic value of 0.30. One potential explanation for this failure is that there are insufficient negative proportional effect scores at low baseline performance levels. As a result, the model fails to adequately capture this section of the observed function (cf. **Figure 1**). However, it appears that these negative proportional effects do affect the overall function in a different way, essentially by lowering the asymptote value. In essence these functions are more linear (and negatively shifted down the *Y*-axis) than one would ideally want in order to recreate the function shown in **Figure 1**.

In contrast, when a floor that is greater than zero is employed (**Figures 5B,C**), the functions much more clearly mirror that shown in **Figure 1**; the larger floor value giving rise to fewer very negative proportional effect scores at low levels of baseline recall. Furthermore, the greater the noise associated with the estimates of baseline and referent performance, the longer the function takes to reach its asymptotic value. Importantly, in panels B and C the functions do converge on the same asymptote value, corresponding to a 30% proportional cost of the manipulation.

## Discussion

The first aim of this paper was to present a series of simulations of children’s potential short-term memory performance under different experimental conditions. This was done in order to test whether a proportional scaling model can effectively account for the pattern of data seen in developmental studies, and to explore what additional assumptions might be needed to adequately capture the full range of performance observed at all ages. The second aim was to provide an indication of the ways in which future empirical research in this area might take proportional scaling into account, and how developmental data might be best measured and modeled in such studies.

Although there is previous evidence to suggest that manipulations such as the phonological similarity effect do scale proportionally (Logie et al., 1996; Beaman et al., 2008; Jarrold and Citroën, 2013), it also appears that such effects are smaller than, and sometimes even in the opposite direction to, those predicted by a simple proportional scaling account among very young children (Jarrold and Citroën, 2013). Indeed, the preliminary re-analysis of the Jarrold and Citroën (2013) data presented briefly above showed that the developmental change in the size of the proportional phonological similarity effect could be modeled by a single mathematical function, namely a negative exponential growth function. We do not wish to argue that this particular function necessarily provides a better fit to existing data than, say, a power law relation. However, it does capture the fact that proportional effect sizes range from small positive magnitudes to large negative magnitudes when baseline levels of recall are low, but are positive and constant at higher levels of baseline performance. Note also that here, and throughout, we are using baseline level of recall as a proxy for an individual’s developmental level.

As noted, the starting point for these simulations was the suggestion that the size of these manipulation effects in verbal short-term memory might be proportional; that is, the absolute size of the difference between recall of easy (short or phonologically dissimilar words) and difficult (long or phonologically similar words) items would be proportional to overall level of recall. There are good theoretical reasons for making this assumption in the context of studies of the word length and the phonological similarity effect. If the word length effect is caused by the greater interference that results from longer words containing more phonemes (cf. Brown and Hulme, 1995; Lewandowsky and Oberauer, 2008), then the size of this effect would be expected to scale with the number of such words held in memory. Similarly, the phonological similarity effect necessarily depends on the overlap between items within the just-presented list; the greater the number of these items, the greater one would expect the absolute effect of similarity to be (cf. Page and Norris, 1998; Baddeley, 2012).

However, as **Figure 2** shows, a simple proportional scaling model fails to replicate the pattern shown in **Figure 1**, because it necessarily fails to reproduce the negative proportional manipulation effects that are seen at lower levels of baseline recall. Simulation 2 showed that assuming noise in the data whose spread was proportional to an individual’s level of overall recall also failed to produce a function that mirrored that seen in **Figure 1**. One might well expect noise to scale in line with overall level of performance, because as baseline performance increases so there is a greater range over which performance in the harder manipulation condition might range, giving rise to the often observed statistical phenomenon of heteroscedasticity. However, while such noise necessarily leads to observed heteroscedasticity when plotting absolute scores (see **Figure 3A**), the fact that noise was centered around zero and was drawn from a distribution whose spread was proportional to baseline recall means that its effects are symmetrical and constant when the size of the overall manipulation effect is coded proportionally (see **Figure 3B**).

Instead, assuming that the spread of the noise distribution remains constant over all levels of recall (**Figure 4B**) did provide a simulated dataset that matched that seen in **Figure 1**. This is because noise of a constant spread has the same *absolute* effect on all predicted scores, but a relatively greater influence on small than on large predicted scores when these are proportionalised (i.e., random variation of plus or minus 2 partial credit score points has a greater proportional effect on predicted scores of 4 than on predicted scores of 8). In addition, noise of a constant magnitude can lead to performance in the harder manipulation condition dropping to floor (which in the case of Simulation 3 was a partial credit score of 0.01). This imposes an upper limit on the size of the absolute manipulation effect that equals the level of performance in the baseline condition (baseline score – 0.01, see **Figure 4A**). This, in turn, necessarily limits the proportional size of the manipulation effect ([baseline score – 0.01]/baseline score, which tends to 1, see **Figure 4B**). In contrast, there is no equivalent lower limit to either absolute or proportional measures of the manipulation effect. This is because when noise randomly increases the observed harder manipulation condition score beyond that predicted by proportional scaling, then this only leads to negative absolute and proportional manipulation effects. Indeed, when baseline scores are particularly low, proportional scoring produces large negative effects at these low levels of recall because negative absolute effects are then divided by small baseline values.

This raises the question of whether one should plot proportionalised effect scores in the first place; if statistical artifacts affect this function at low baseline levels, does this provide an appropriate way of representing the data? We would argue that proportionalising is appropriate, and in fact produces a more comprehensible representation of the data than a plot of absolute scores. As **Figure 4A** shows, the problem of noise producing floor values for the observed harder manipulation condition score means that one cannot simply fit a linear regression through the absolute measure of the effect and expect to produce a function whose slope equals the proportional cost of the manipulation (which otherwise would be a sensible approach, see **Figure 2A**). In contrast, although the interpretation of proportional effects at low levels of recall is somewhat complicated, a plot of proportional effects does produce a function that levels off to indicate the proportional cost of the manipulation (see **Figure 4B**, and also Logie et al., 1996).

Indeed, Simulation 4 showed that by plotting proportionalised effects one can clearly reveal the influence that variations in the spread in the noise distribution and in the level of floor performance has on these functions. In particular, a larger spread in the noise distribution produces functions that level off at relatively higher levels of baseline performance for the reasons outlined above. In addition, in these simulations a floor of essentially zero did not produce curves that mirrored that seen in **Figure 1**. In this case, although the underlying distribution of data points was necessarily similar to that shown in **Figure 4B**, there were insufficient negative scores in these simulations to allow for a negative growth function to fit the data in the way that it should. Instead negative growth functions fitted the data more appropriately when floor scores were higher, by virtue of producing relatively more negative proportionalised effects at higher levels of baseline recall. Again we would argue that a ‘functional floor’ above zero is not an unreasonable assumption. If one is manipulating either word length or phonological similarity then there will be a list length below which this manipulation would not be expected to be effective – for example, phonological similarity necessarily cannot occur with only one item in the list. In addition, one might expect individuals to be able to recall at least one item from any list, however long the list and however long or confusable the memoranda, by virtue of just maintaining the last-presented item.

The other assumption that one might question, and which underpins the successful simulations represented in **Figures 4B** and **5**, is that of a noise distribution whose spread remains constant in size across all levels of performance. As noted above, one might instead expect the size of any noise distribution to scale with level of performance. One point to note in response is that one could potentially produce a simulation that combined two noise distributions, one whose spread was constant and another whose spread scaled with baseline performance. Such a simulation is not reported here, partly for the sake of clarity, and also because it is not obvious what it would add to our arguments. We have shown that noise with constant spread is necessary to reproduce the observed data. However, there is every reason to suspect that a combined model that also included noise with proportional spread would produce a broadly similar pattern, provided that the relative balance of the two noise effects was not weighted too heavily in favor of the latter. The second point to highlight is that there are reasons for thinking that noise with a constant spread would be associated with verbal short-term memory tasks of the form employed here. Specifically, most developmental studies use a span procedure to measure verbal short-term memory capacity. This involves presentation of a set number of trials at each list length, beginning at short list lengths and stopping when the participant fails to recall trials correctly at a particular upper list length. Although the total number of trials that a participant receives will therefore vary, depending on their verbal short-term memory span, all participants receive the same number of trials at their upper list length. If one assumes that all participants can recall all trials on list lengths below or up to their span, but are unable to recall all of the items on all trials at list lengths above their span, then any noise in the estimate of their memory capacity will come from the fixed number of trials that are presented at the final, highest list length that they receive. This will particularly be the case when using partial credit scoring. A further reason why noise might have a constantly sized distribution reflects the measurement error that comes from that the fact that performance is not measured on a continuous scale, but instead necessarily increments in discrete steps. There are of course other threats to validity that can cause noise in experiments, see Shadish et al. (2002) for an extensive list, but we think that these are the most relevant to our type of simulation.

To summarize the above points in the context of the first aim of this paper, two of the simulations (Simulations 3 and 4) produce a pattern of data that matches that observed by Jarrold and Citroën (2013). This shows that such data can be generated by making three simple assumptions. The first is that a manipulation (which could be of word length or of phonological similarity) produces effects that are proportional in size to overall level of recall. The second is that the measurement of the data points that underpin these effects is subject to noise whose distribution has a constant spread across levels of performance. The third, which is arguably less crucial but which may be important in some data sets, is that there is a floor to performance below which recall levels cannot drop. We have argued above that these assumptions are reasonable ones to make in the context of studies of children’s verbal short-term memory.

Turning to our second aim, we would therefore expect that other researchers working in this area would generate data that match those shown in **Figure 1**, and that could also be fit with negative exponential growth functions of the form used in **Figures 1** and **5** if coded in terms of proportionalised word length or phonological similarity effects. Importantly, this paper has shown that such plots do not produce the flat functions that one would intuitively expect, because of the noise that is necessarily associated with any single measurement. However, the functions that are obtained are comprehensible, and can be interpreted; in particular, the asymptotic level of the function provides an index of the proportional cost of the manipulation that one would expect once performance is sufficiently above floor.

Crucially, plotting developmental data in this way provides a means of testing the widely held view that children’s use of rehearsal undergoes a qualitative change in middle childhood. Being able to account for the development of a manipulation effect with a single mathematical function casts doubt on the suggestion of a qualitative change in the underlying cognitive processes that give rise to this effect. We accept that some authors would argue strongly that the word length effect does not necessarily index rehearsal but rather reflects forgetting due to interference, and that the phonological similarity effect for visually presented material only measures an individual’s ability to recode, which is not necessarily the same as rehearsal (see Jarrold and Hall, 2013). However, as reviewed above, many others do argue that these effects index rehearsal processes. The literature is also replete with claims that the absence of these effects in young children shows that rehearsal undergoes a qualitative change around the age of 6 or 7 years. Our simulations show that these assumptions are not necessary to produce the observed developmental pattern, and suggest ways in which the development of verbal short-term memory effects should be plotted and examined in future.

## Conflict of Interest Statement

The Guest Associate Editor, Martin Lehmann, declares that, despite having collaborated with author, Christopher Jarrold, the review process was handled objectively and no conflict of interest exists. The authors declare that the research was conducted in the absence of any commercial or financial relationships that could be construed as a potential conflict of interest.
